# Cas9/gRNA targeted excision of cystic fibrosis-causing deep-intronic splicing mutations restores normal splicing of *CFTR* mRNA

**DOI:** 10.1371/journal.pone.0184009

**Published:** 2017-09-01

**Authors:** David J. Sanz, Jennifer A. Hollywood, Martina F. Scallan, Patrick T. Harrison

**Affiliations:** 1 Department of Physiology, BioSciences Institute, University College Cork, Cork, Ireland; 2 School of Microbiology, University College Cork, Cork, Ireland; International Centre for Genetic Engineering and Biotechnology, ITALY

## Abstract

Cystic Fibrosis is an autosomal recessive disorder caused by mutations in the *CFTR* gene. CRISPR mediated, template-dependent homology-directed gene editing has been used to correct the most common mutation, c.1521_1523delCTT / p.Phe508del (F508del) which affects ~70% of individuals, but the efficiency was relatively low. Here, we describe a high efficiency strategy for editing of three different rare *CFTR* mutations which together account for about 3% of individuals with Cystic Fibrosis. The mutations cause aberrant splicing of *CFTR* mRNA due to the creation of cryptic splice signals that result in the formation of pseudoexons containing premature stop codons c.1679+1634A>G (1811+1.6kbA>G) and c.3718-2477C>T (3849+10kbC>T), or an out-of-frame 5’ extension to an existing exon c.3140-26A>G (3272-26A>G). We designed pairs of Cas9 guide RNAs to create targeted double-stranded breaks in *CFTR* either side of each mutation which resulted in high efficiency excision of the target genomic regions via non-homologous end-joining repair. When evaluated in a mini-gene splicing assay, we showed that targeted excision restored normal splicing for all three mutations. This approach could be used to correct aberrant splicing signals or remove disruptive transcription regulatory motifs caused by deep-intronic mutations in a range of other genetic disorders.

## Introduction

Cystic fibrosis (CF) is a chronic and progressive disorder affecting more than 70,000 people worldwide, characterised by dysfunctional secretory epithelial cells which cause obstructions in the lung airways and pancreatic ducts [1]. The disease is caused by mutations in both alleles of the *CFTR* gene, which encodes an apical membrane Cl^-^/HCO_3_^-^ channel [2–4]. Of the 2019 sequence variants identified in the CFTR gene to date (www.genet.sickkids.on.ca/Home.html), at least 281 have been characterised as disease-causing [www.cftr2.org; 5], and have been stratified into five or six different classes according to disease severity and the molecular basis by which they disrupt ion channel activity [6,7]. To date, small molecule drugs have been developed and approved for 34 different CF-causing mutations [8–12], including the most common CF-causing mutation F508del found in 70% of individuals with CF [4,5]. However, this leaves ≥247 disease-causing mutations for which specific treatments are not available.

One potential therapeutic option for all these mutations is cDNA-based gene therapy. More than 20 years of clinical trials have established the safety of DNA delivery to the lungs of individuals with CF, but the best clinical outcome reported date is a small but significant stabilisation of lung function during a multiple-dose cDNA protocol [13]. Another option under investigation is the use of gene editing to correct CF-causing mutations. Most studies to date have focused on precision gene editing using genome-specific nucleases such as CRISPR/Cas9 to create a targeted double stranded break (DSB) in the genome close to the mutation, then using a donor or template DNA molecule to provide the necessary sequence information to correct the mutation by the homology directed repair (HDR) pathway [14,15]. In principle, this approach could be adapted for any individual CF-causing mutation, or even many different mutations [16], but a number of factors, not least the relatively low efficiency of editing potentially mitigate the development of a HDR-based approach for therapeutic development.

In this study, we chose to investigate the use of a CRISPR/Cas9-based non-homologous end joining (NHEJ) approach to edit a small number of CF-causing mutations in a significantly different way. NHEJ is the default pathway for repair of double-stranded breaks (DSBs) which occurs at all stages of the cell cycle and typically results in higher frequency repair events than HDR [17,18]. The obvious limitation is that repair of a single DSBs by NHEJ typically results in disruption of the target site by indel formation, so is normally used for gene knockout studies, rather than repair of disease-causing mutations. However, based on the observation that NHEJ repair of two DSBs results in target deletion of the genomic sequence between the two Cas9/gRNA target sites [19], we wondered if this approach could be used to excise a small number of CF causing mutations which lie in deep intronic regions of *CFTR*.

The three mutations we studied affect approximately 1.5% of individuals with CF and do not disrupt the classical canonical splicing regions [20], rather they cause CF by creating strong alternative splice sites which result in formation of pseudoexons or extend existing exons in *CFTR*. The first mutation, c.1679+1634A>G, creates a very strong splice acceptor in intron 12 of *CFTR* with 99% of transcripts containing a 49bp pseudoexon with an in-frame TAA stop codon in the pseudoexon causing premature termination of the CFTR protein [21], and is classified as a severe CF-causing mutation. The second, c.3140-26A>G, creates a splice acceptor site 26 bp before exon 20 and therefore extends this exon by 25bp [22]. The resultant frameshift leads to premature termination of CFTR at a TGA stop codon. Whilst c.3140-26A>G disrupts 95% of *CFTR* transcripts, the production of ~5% normal transcripts is thought to explain the milder CF phenotype observed in individuals with this mutation. The final mutation, c.3718-2477C>T, creates a splice donor deep within intron 22 and leads to inclusion of an 84 bp pseudoexon (which contains an in-frame TAA stop codon) into the mRNA [23]. For individuals with c.3718-2477C>T, the 10^th^ most common CF allele [5], a wide range of aberrant splicing has been reported, with a strong correlation between the level of mis-splicing and severity of disease [24–26].

For each of the three mutations, we showed that CRISPR Cas9/gRNA pairs could be used to successfully excise them via a non-homologous end joining (NHEJ) pathway. NHEJ-mediated targeted excision occurred in ≥25% of transfected cells, a level of editing which is 10-fold higher than our previous study of HDR-based editing with Cas9/gRNA in the same locus in the same cells [18]. Moreover, the NHEJ-editing approach restored normal splicing to a level equal to, or exceeding the level of targeted excision for all three mutations.

## Materials and methods

### CFTR nomenclature

All CFTR variants are named according to the CFTR2.org database [5] and NM000492.3 sequence, with the legacy name for each of the three deep-intronic mutation shown in parentheses upon first time use.

### gRNA design and cloning

The CRISPR design tool (http://crispr.mit.edu/) was used to identify putative gRNAs sequences in a region of ~300bp centred on the three selected intron mutations in the *CFTR* gene. Oligos encoding the selected gRNAs (see S1, S2, S3 and S5 Figs) were converted to dsDNA fragments then cloned into the pSp-Cas9-(BB)-2A-GFP vector (addgene.org PX458) by GoldenGate cloning [27]. The GFP expressed from this vector is subsequently used to sort cells that express Cas9 and gRNAs (see below). To express both an upstream and downstream gRNA in a single vector, the primers pcas9TANDfw and pcas9TANDrv were used to amplify the U6 promoter/gRNA from the downstream vector and cloned into the *Xba*I site located after the upstream gRNA expression cassette of the upstream gRNA expressing plasmid.

### Cell lines, transfections and FACS sorting

The cystic fibrosis tracheal epithelial (CFTE) cell line [28] is immortalized and of human origin, and is homozygous for the c.1521_1523delCTT mutation (p.Phe508del) and was obtained from Dieter Gruenert (Department of Otolaryngology–Head and Neck Surgery, UCSF, San Francisco, CA) and maintained in modified Eagle's medium (supplemented with 10% foetal calf serum, 1% L-glutamine, and 1% penicillin and streptomycin; Sigma-Aldrich, Arklow, Ireland) and incubated at 37°C and 5% CO_2_; CFTE cells were used to evalute genomic activity of Cas9/gRNAs. HEK293T cells [29] were cultured in Dulbecco modified Eagle's medium (supplemented with 10% foetal calf serum, 1% L-glutamine, and 1% penicillin and streptomycin; Sigma-Aldrich, Arklow, Ireland) and incubated at 37°C and 5% CO_2_; HEK293T cells were used for all mini-gene experiments. For transfection, 100,000 cells were seeded on 24 well plates and 24 hrs prior to transfection with a total of 1 μg plasmid DNA and 4 μl Lipofectamine^™^ 3000 (Invitrogen, Dun Laoghaire, Ireland) following the manufacturer’s recommended protocol. 48h after transfection cells expressing GFP were sorted using a FACSAria II system (BD Biosciences, Oxford, UK), the selected cells were grown for an additional 72h before total DNA was extracted using DNAeasy kit (Qiagen, Manchester, UK). PCR and agarose gel electrophoresis were performed using specific primers to detect the ability of expressed gRNAs pairs to produce deletions of the expected size in CFTE genomic DNA.

### Minigene construction

Target regions were amplified from genomic DNA from human lung tissue DNA (Genomic DNA—Human Adult Normal Tissue Lung, from a single donor, Amsbio, Abingdon, UK) using PfuUltra High-Fidelity DNA polymerase (New England Biolabs, Stillorgan, Ireland) and primers containing a 5′-tail with a restriction site for *Xho*I, *Nhe*I, or *Xba*I (S5 Fig). Target amplicons were sub-cloned in *Xho*I/*Nhe*I sites in a modified version (3053G>A to destroy a cryptic splice site) of the exon-trapping vector pSPL3 (Invitrogen, Dun Laoghaire, Ireland). To confirm the fidelity of the cloned sequences, plasmids were sequenced (Eurofins, Ebersberg, Germany) using primers SEQpspfw and SEQpsprv. To create mini-gene plasmids containing the CF-causing mutations, site-directed mutagenesis was performed using the QuikChange mutagenesis kit (Agilent, Cork, Ireland) and oligonucleotides as shown in S5 Fig.

### Minigene assay

Minigene plasmids containing wild type or deep-intronic mutant sequences were transfected into HEK293T cells in the presence of either a pTandem plasmid expressing Cas9 and two gRNAs, or a a negative control plasmid, pcDNA3.1+ (InVitrogen, Dun Laoghaire, Ireland).

To analyse transcripts, total RNA was extracted and purified 48 hours post-transfection using a Nucleospin-RNA-II kit (Macherey-Nagel, Düren, Germany). Total RNA from each sample was reverse-transcribed using the GoScript Reverse Transcription system (Promega, Southampton, UK), and minigene-specific transcripts amplified by PCR using with GoTaq^®^ Hot Start Polymerase (Promega, Southampton, UK) with primers pspRTfw and pspRTrv (S5 Fig). For fragment length analysis (Eurofins, Ebersberg, Germany), samples were amplified with FAM-labelled forward primer and unlabelled reverse primer.

To analyse targeted deletions, DNA was extracted and purified 48 hours post-transfection using a DNAeasy kit (Qiagen, Manchester, UK). DNA from each sample was amplified by PCR using with GoTaq^®^ Hot Start Polymerase (Promega, Southampton, UK). Deletions in MG-12 plasmids were detected with primers pspSEQFw and in12Drv, deletions in MG-19 plasmids were detected with in19Ufw and pspSEQrv, and deletions in pspSEQFw and in22Drv. Band intensity was measured by densitometry. Efficiency of targeted deletion was expressed as the densitometric value of the lower band divided by the sum of the densitometric values of the lower and upper bands, multipled by 100%. Where required, PCR and RT-PCR bands were gel purified from agarose gels and sequenced (Eurofins, Ebersberg, Germany).

## Results

### Design and screening of gRNA pairs to target deep-intronic mutations in *CFTR*

For each of the three deep-intronic mutations, multiple gRNAs were designed either side of the CF-causing single nucleotide substitution to create a deletion of ≥50bp. For the c.1679+1634A>G mutation in intron 12, three upstream gRNAs (designated 12U1 to 12U3) and three downstream gRNAs (12D1 to 12D3) were combined in pairs to test nine different gRNA permutations (S1a Fig). Two of nine gRNAs pairs tested created a deletion of the expected size in *CFTR* with an efficiency of 28–33% (S1b Fig). For the c.3140-26A>G mutation in intron 19, two upstream gRNAs (19U1 and 19U2) cutting upstream and two downstream gRNAs (19D1 and 19D2) were combined in pairs to test four different gRNA permutations (S2a Fig). One of the pairs created a deletion of the expected size in *CFTR* with an efficiency of 30% (S2b Fig). Finally, for the c.3718-2477C>T in intron 22, three upstream gRNAs (22U1 to 22U3) and two downstream gRNAs (22D1 and 22D2) were also combined in pairs to test six different gRNA permutations (S3a Fig). Four of the pairs created deletions of the expected size in *CFTR* with an efficiency of 34–47% (S3b Fig).

Having identified the most efficient pair of gRNAs to excise each mutation, we then created three plasmids which allow co-expression of these gRNA pairs in tandem from the same plasmid with Cas9 (pTandem-in12, pTandem-in19, and pTandem-in22). Each of these constructs was shown to edit their respective intronic regions in CFTE cells at similar efficiency to when used as two separate plasmids (S4a and S4b Fig), and the pTandem clones were used in subsequent experiments described below. Sequence analysis of PCR product generated from the intron 22 (Fig 3f) which have undergone targeted excision by Cas9/gRNAs encoded by pTandem-in22 show a roughly equal mix of three slightly different but precise end joining events which are consistent with the DSB created by Cas9 being either 3 or 4 nucleotides upstream of the PAM sequences of the two gRNA target sites (S4c Fig).

### Targeted excision of CF-causing mutations is highly efficient and rescues splicing defects

In order to test if targeted deletion of the CF-causing mutations could rescue splicing, we created a mini-gene reporter vector for each target sequence and measured the splicing pattern with and without co-transfection of the respective pTandem Cas9/gRNA expression vector. The mini-gene approach has been used to characterise splicing of human genes such as BRCA1/2 [30], and is established as a robust tool for clinical interpretation of *CFTR* splice sites [31].

#### c.1679+1634A>G in intron 12

**Fig 1:**
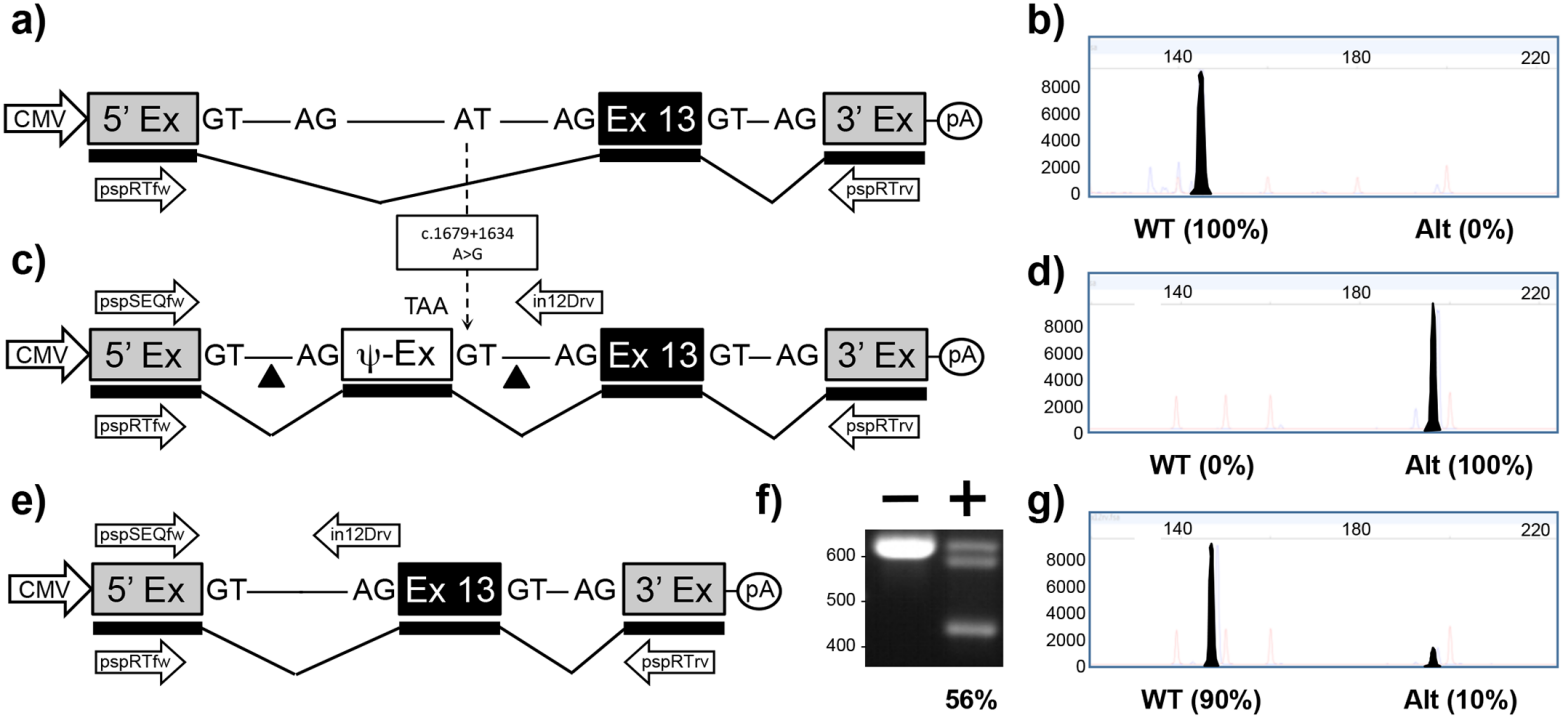
Mini-gene analysis of c.1679+1634A>G mutation. a) Schematic of pMG-in12-WT plasmid comprising a contiguous ~1.5kb region of *CFTR* containing part of intron 12, exon 13 and part of intron 13 cloned between the 5’ and 3’ exons of the pSPL3 vector. Exons are shown as boxes, introns as lines, and predicted transcript as thicker line shown underneath. Arrows below transcript represent pspRTfw and pspRTrv primers for RT-PCR. b) Electropherogram analysis of RT-PCR products (filled peaks) generated from HEK293T cells transfected with pMG-in12-WT and size markers (unfilled peaks). c) Schematic of pMG-in12-A>G mutation which differs from pMG-in12-WT by the A>G change shown with dotted arrow. The A>G change creates a pseudo exon (Ψ-EX) which contains an in-frame stop codon (TAA). The triangles flanking the Ψ-EX indicate target sites of Cas9/gRNAs encoded by pTandem-in12 vector. Arrows above DNA represent pspSEQfw and in12Drv primers for PCR. d) Electropherogram analysis of RT-PCR products generated from HEK293T cells transfected with pMG-in12-A>G. e) Schematic of pMG-in12-A>G mutation following targeted excision and repair. f) Agarose gel electrophoresis analysis of targeted deletions in pMG-in12-A>G measured by PCR. “-” lane is PCR products generated from HEK293T cells transfected with pMG-in12-A>G only, “+” lane is PCR products generated from HEK293T cells transfected with pMG-in12-A>G and pTandem-in12. The top band is generated from untargeted DNA, the bottom band is generated from DNA containing the targeted deletion, and the middle band is most likely heteroduplexes formed during the late stages of PCR; similar bands have been reported during PCR analysis of the CF-causing c.1521_1523delCTT deletion and verified as heteroduplexes [32]. g) Electropherogram analysis of RT-PCR products generated from HEK293T cells co-transfected with pMG-in12-A>G and pTandem-in12 vector.

Transfection of the pMG-in12-WT plasmid in HEK293T cells resulted in a single RT-PCR product confirming that the 5’ exon has spliced accurately to *CFTR* exon 13 which in turn splices accurately to the 3’ exon (Fig 1b and S1c Fig). In contrast, when cells were transfected with the pMG-in12-A>G, a modified version of the plasmid which contained the c.1679+1634A>G mutation (Fig 1c), there was 100% incorporation of the 49 bp pseudoexon (Fig 1d and S1d Fig). Co-transfection of pMG-in12-A>G with pTandem-in12 resulted in a high level of 56% targeted deletions (Fig 1e) as measured by PCR (Fig 1f), which resulted in rescue of splicing to 90% of normal levels (Fig 1g and S1e Fig).

#### c.3140-26A>G in intron 19

**Fig 2:**
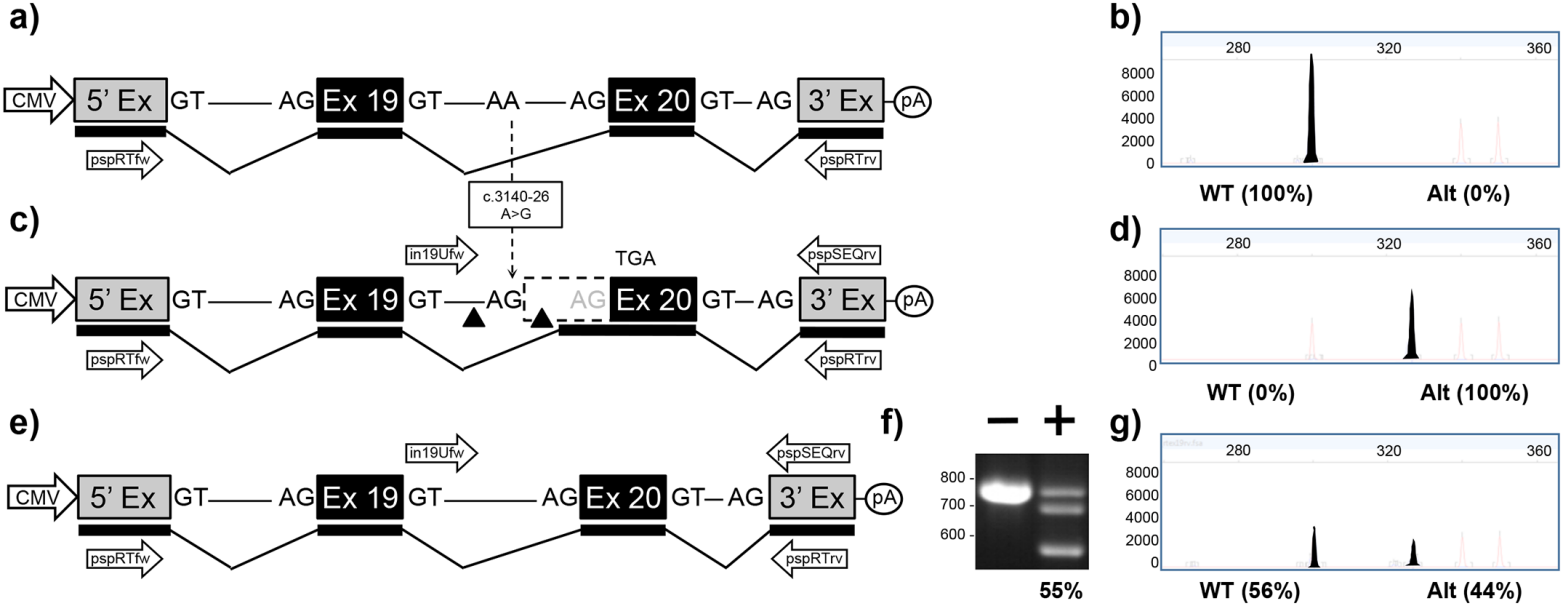
Mini-gene analysis of c.3140-26A>G mutation. a) Schematic of pMG-in19-WT plasmid comprising a contiguous ~2.0 kb region of *CFTR* containing part of intron 18, exon 19, all of intron 19, exon 20 and part of intron 20 cloned between the 5’ and 3’ exons of the pSPL3 vector. b) Electropherogram analysis of RT-PCR products generated from HEK293T cells transfected with pMG-in19-WT. c) Schematic of pMG-in19-A>G mutation which differs from pMG-in19-WT by the A>G change shown with dotted arrow. The A>G change extends exon 20 by 25 bp which shifts the reading frame and eventually results in-frame stop codon (TGA). The triangles indicate target sites of Cas9/gRNAs encoded by pTandem-in19 vector. Arrows above DNA represent in19Ufw and pspSEQrv primers for PCR. d) Electropherogram analysis of RT-PCR products generated from HEK293T cells transfected with pMG-in19-A>G. e) Schematic of pMG-in19-A>G mutation following targeted excision and repair. f) Agarose gel electrophoresis analysis of targeted deletions in pMG-in19-A>G measured by PCR. “-” lane is PCR products generated from HEK293T cells transfected with pMG-in19-A>G only, “+” lane is PCR products generated from HEK293T cells transfected with pMG-in19-A>G and pTandem-in12. g) Electropherogram analysis of RT-PCR products generated from HEK293T cells co-transfected with pMG-in19-A>G and pTandem-in19 vector.

Transfection of the pMG-in19-WT plasmid in HEK293T cells resulted in a single RT-PCR product confirming that the 5’ exon has spliced accurately to *CFTR* exon 20 which in turn splices accurately to the 3’ exon (Fig 2b and S2c Fig). In contrast, when cells were transfected with the pMG-in19-A>G, a modified version of the plasmid which contained the c.3140-26A>G mutation (Fig 2c), exon 20 was extended by 25 bp in 100% of transcripts (Fig 2d and S2d Fig). Co-transfection of pMG-in19-A>G with pTandem-in19 resulted in a high level of 55% targeted deletions (Fig 2e) as measured by PCR (Fig 2f), which resulted in rescue of splicing to essentially the same level (Fig 2g and S2e Fig). Of note, the c.3140-26A>G mutation has previously been studied in a mini-gene construct, and also resulted in 100% aberrant splicing pattern as shown here, even though this mutation gives rise to ~5% normal transcripts in nasal epithelial cells [22,33].

#### c.3718-2477C>T in intron 22

**Fig 3:**
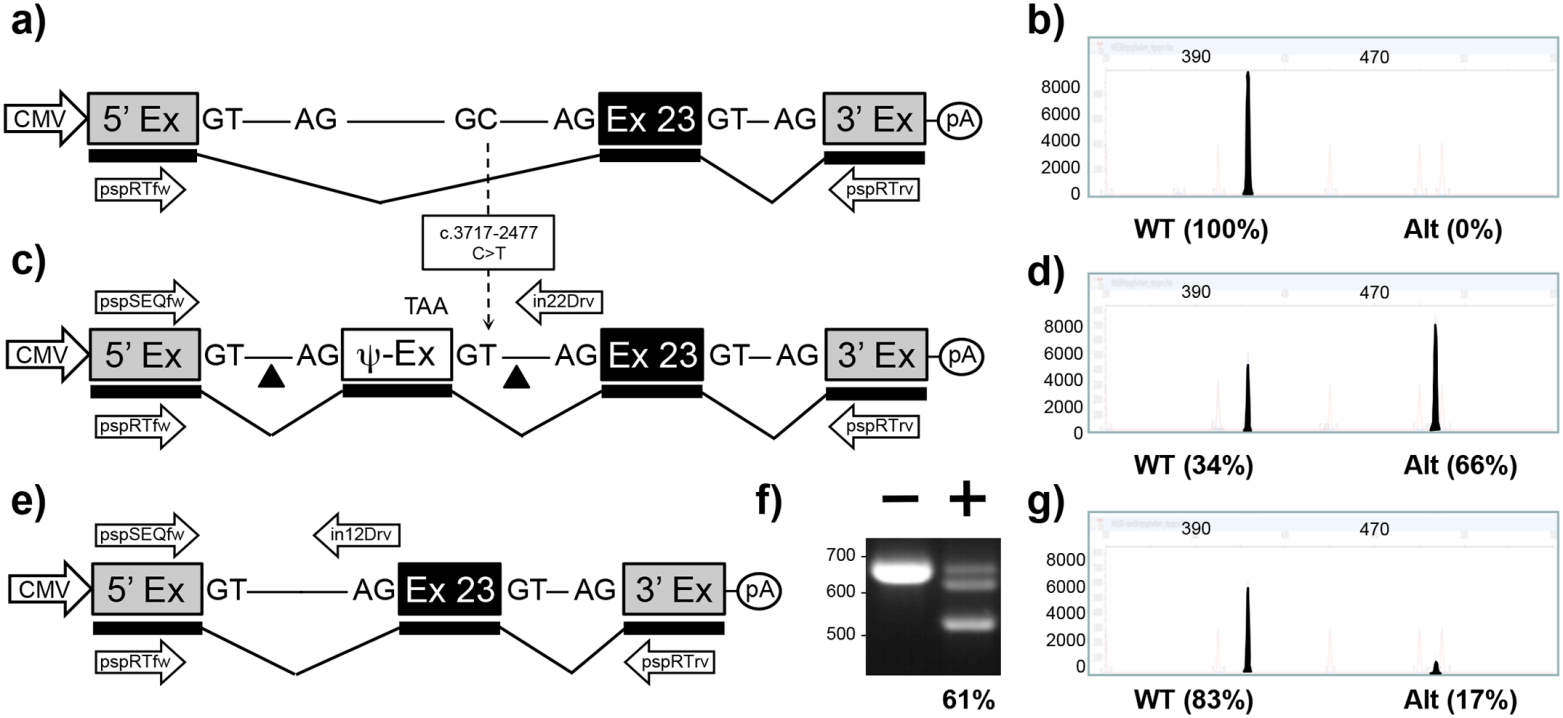
Mini-gene analysis of c.3718-2477C>T mutation. a) Schematic of pMG-in22-WT plasmid comprising a contiguous ~3.2 kb region of *CFTR* containing part of intron 22, exon 23, and part of intron 23 cloned between the 5’ and 3’ exons of the pSPL3 vector. b) Electropherogram analysis of RT-PCR products (filled peaks) generated from HEK293T cells transfected with pMG-in22-WT. c) Schematic of pMG-in22-C>T mutation which differs from pMG-in22-WT by the C>T change shown with dotted arrow. The C>T change creates a pseudo exon (Ψ-EX) which contains an in-frame stop codon (TAA). The triangles flanking the Ψ-EX indicate target sites of Cas9/gRNAs encoded by pTandem-in22 vector. Arrows above DNA represent pspSEQfw and in22Drv primers for PCR. d) Electropherogram analysis of RT-PCR products generated from HEK293T cells transfected with pMG-in22-C>T. e) Schematic of pMG-in22-C>T mutation following targeted excision and repair. f) Agarose gel electrophoresis analysis of targeted deletions in pMG-in22-C>T measured by PCR. “-” lane is PCR products generated from HEK293T cells transfected with pMG-in22-C>T only, “+” lane is PCR products generated from HEK293T cells transfected with pMG-in22-C>T and pTandem-in22. g) Electropherogram analysis of RT-PCR products generated from HEK293T cells co-transfected with pMG-in22-C>T and pTandem-in22 vector.

Transfection of the pMG-in22-WT plasmid in HEK293T cells resulted in a single RT-PCR product confirming that the 5’ exon has spliced accurately to *CFTR* exon 23 which in turn splices accurately to the 3’ exon (Fig 3b and S3c Fig). In contrast, when cells were transfected with the pMG-in22-C>T, a modified version of the plasmid which contained the c.3718-2477C>T mutation (Fig 3c), 66% of transcripts incorporate the 84 bp pseudoexon (Fig 3d and S3d Fig). Co-transfection of pMG-in22-C>T with pTandem-in12 resulted in a high level of 61% targeted deletions (Fig 3e) as measured by PCR (Fig 3f). Which resulted in a 4-fold reduction of aberrant splicing to just 17% (Fig 3g and S3e Fig). A previous study using a similar c.3718-2477C>T mini-gene construct reported that all transcripts were mis-spliced [26]. However, the previous study was performed in COS-1 and NIH-3T3 cells, and a low level of normal splicing was observed when these cells were transiently transfected with a plasmid encoding E4-ORF6 from Adenovirus type 5 [34]. Given our mini-gene studies where performed in HEK293T cells which are stably transformed with the fragments from the Adenovirus type 5 genome [35], then this may account, at least in part, for the difference.

## Discussion

Proof-of-principle for correction of *CFTR* mutations by gene editing was originally demonstrated using small short fragment homologous recombination (SFHR) in cells [36,37], and mouse lung [38], albeit at low efficiency. Of the subsequent studies reporting HDR-mediated correction of CF-causing mutations using a variety of programmable and RNA-guided nucleases approaches [15], the highest level of repair reported was 9.2% in a CF bronchial epithelial cell line using triplex-forming peptide nucleic acids and donor DNA delivered by nanoparticles [39]. Using the same reagents, this translated into 1.2% editing of mouse lung as measured by deep sequencing [39].

Based on our previous observation that NHEJ-mediated editing of DSBs created by Cas9/gRNA [18] and ZFNs [17] occurs at a substantially higher frequency than HDR, we wondered if an NHEJ-based approach could be used to correct any CF-causing mutations. Given that Cas9/gRNA pairs can create targeted deletions within the genome equivalent to the region between the two DSBs generated by NHEJ repair [19], we postulated that this approach could be used to efficiently excise selected CF-causing mutations located in intron regions, which lie outside of the classical canonical splicing signals [20]; we studied three of these mutations [21–23]. Once the CF-causing region was excised, we predicted that normal splicing should be restored.

For two of these mutations, c.1679+1634A>G and c.3718-2477C>T, the deep-intronic location meant that there were essentially no restrictions on selection of upstream and downstream gRNA target sites. For the c.1679+1634A>G and c.3718-2477C>T mutations, targeted excision resulted in a substantially higher level of splicing restoration compared to the level of excision. This is most likely a consequence of indels created at the DSB created by the gRNA closest to the mutation in instances were the two gRNAs do not result in an excision event.

However, for the c.3140-26A>G mutation which is located just upstream of the splice acceptor in intron 19 there were only two possible downstream gRNA sites, both of which create DSBs within the canonical splice acceptor site. Of these two gRNAs, only one created an efficient target excision when used as a pair with an upstream gRNA. The targeted excision event predicted to occur with these oligos should create a splice acceptor with a hybrid 13/21 base polypyrimidine tract which has the same pyrimidine composition as the original splice acceptor (see S2a Fig).

The overall efficiency of Cas9/gRNA targeted excision by NHEJ for the three mutations ranged from 25–47% of transfected cells, roughly 10- to 20-fold higher than our previous study of Cas9/gRNA HDR in the same locus in the same cells [18]. It is obviously important to test this approach in an *in vivo* model of CF, but this is not currently possible due to sequence differences between species. One option is to redesign the gRNAs to target *Cftr* from other species as done with the PNA-HDR approach [39], though it may be more informative to study in humanised animal models of CF as they become available. Such *in vivo* studies would also be required to determine the percentage of cells that need to be rescued in order to improve or preserve lung function. *Ex vivo* studies have shown that mixed populations of lung epithelia cells where as little as 6–10% of the population express *CFTR* show the same overall level of Cl- efflux via the CFTR ion channel [40], whereas 25% of the population of cells may be required to restore normal airway surface liquid height [41].

The simplicity of the Cas9/gRNA targeted excision approach, specifically that a donor template is not required, offers a number of delivery routes for potential clinical use. The most recent *CFTR* gene therapy trial established that repeat dosing of the human lung with plasmid DNA is safe (Alton et al., 2015], and the plasmid used herein is of a similar size to pGM169 used in that study. It may also be feasible to deliver the Cas9/gRNA as a ribo-protein complex [42–44], or simply as RNA molecules (gRNAs and Cas9 mRNA); mRNA delivery of TAL-effector nucleases (TALENs) has recently been used to create NHEJ-edited CAR T cells successfully used as a bridging therapy in two patients with B-ALL [45]. Alternatively, the Cas9/gRNA system could be delivered by a number of different virus vectors [46,47] which have been used to successfully delivery *CFTR* in large animal models [48,49], and are in pre-clinical development for a first-in-man CF trial [50]; the CCR5-edited autologous CD4 T cells successfully used to reduce levels of HIV RNA in patients were edited using ZFNs delivered by virus vectors [51].

In summary, the targeted excision approach can successfully and precisely excise small regions of the *CFTR* introns containing disease causing mutations, and targeted excision can restore normal splicing patterns. In its current format, targeted excision is not suitable for CF-causing mutations such as 621+1G->T, 1717-1G->A, 2789+5G->A [5] which disrupt the canonical splice sites, although a number of alternative therapeutic approaches are under investigation for these mutations [52,53]. However targeted excision may be useful for three other mutations in *CFTR* which have been shown to form pseudoexons (c.1584+18672A>G [54] and c.1680-877G>T [55] or extend existing exons (c3717+40A>G [55]. Whilst these six mutations only represent about ~1.6% of individuals with CF, the high efficiency and simplicity of this editing strategy may form the basis of a potential therapeutic approach. The targeted gene excision strategy may also be applicable in the study of more than 75 other genetic disorders where disease-causing deep-intronic mutations have been identified [56].

## Supporting information

S1 Fig c.1679+1634 A>G target region, gRNA selection and mini-gene transcript analysis. **a)** Sequence of 515 bp amplicon generated from intron 12 of CFTR using PCR primers in12Ufw and in12Drv with the 49 bp pseudoexon generated by the A>G mutation shown in UPPERCASE. gRNA target sites are underlined or overlined in blue according to orientation with PAM sequences (shown in red). The target sequences of the two gRNAs used in the pTandem-in12 vector are also shaded in blue. The predicted cut sites for all gRNAs are located 3 bp upstream of the PAM site (open triangles—RuvC site, filled triangles—HNH site). The predicted deletion created by pTandem-in12 is 203 bp. **b)** Agarose gel analysis of PCR amplicons generated from CFTE cells cotransfected with pairs of Cas9/gRNA expression plasmids. **c)** Sequence of main transcripts generated from pMGin12-WT (normal transcript), pMGin12-A>G (pseudoexon inclusion), and pMGin12-A>G co-transfected with pTandem-in12 (pseudoexon exclusion) in HEK293T cells. Arrows indicate junctions between exons.
(TIF)

S2 Fig c.3140-26A>G target region, gRNA selection and mini-gene transcript analysis. **a)** Sequence of 334 bp amplicon generated from intron 19 (lowercase)/EXON 20 (uppercase) using PCR primers inU19fw and in19Drv. gRNA target sites are underlined or overlined in blue according to orientation with PAM sequences (shown in red). The target sequences of the two gRNAs used in the pTandem-in19 vector are also shaded in blue. The predicted cut sites for all gRNAs located 3 bp upstream of the PAM site (open triangles—RuvC site, filled triangles—HNH site). The predicted deletion when using the pTandem-in19 vector is 179 bp. The inset shows predicted polypyrimidine tracts for wild-type intron 19 splice acceptor, c.3140-26A>G intron 19 splice acceptor and c.3140-26A>G intron 19 splice acceptor after targeted deletion. **b)** Agarose gel analysis of PCR amplicons generated from CFTE cells cotransfected with pairs of Cas9/gRNA expression plasmids. **c)** Sequence of main transcripts generated from pMGin19-WT (normal transcript), pMGin19-A>G (pseudoexon inclusion), and pMGin19-A>G co-transfected with pTandem-in19 (pseudoexon exclusion) in HEK293T. Arrows indicate junctions between exons.
(TIF)

S3 Fig 3178-2477C>T target region, gRNA selection and mini-gene transcript analysis. **a)** Sequence of 378 bp amplicon generated from intron 22 of CFTR using PCR primers in22Ufw and in22Drv with the 84 bp pseudoexon generated by the C>T mutation shown in UPPERCASE. gRNA target sites are underlined or overlined in blue according to orientation with PAM sequences (shown in red). The target sequences of the two gRNAs used in the pTandem-in22 vector are also shaded in blue. The predicted cut sites for all gRNAs are located 3 bp upstream of the PAM site (open triangles—RuvC site, filled triangles—HNH site). The predicted deletion created by pTandem-in22 is 141 bp. **b)** Agarose gel analysis of PCR amplicons generated from CFTE cells cotransfected with pairs of Cas9/gRNA expression plasmids. **c)** Sequence of main transcripts generated from pMGin22-WT (normal transcript), pMGin22-A>G (pseudoexon inclusion), and pMGin22-A>G co-transfected with pTandem-in22 (pseudoexon exclusion) in HEK293T. Arrows indicate junctions between exons.
(TIF)

S4 Fig Targeted deletion analysis from pTandem vectors in CFTE cells. a) Deletions in CFTE cells using pTandem vectors anaysed by electropherogram. pTandem-in12 expresses Cas9 plus in12-U2 and in12-D1 gRNAs creating a 203 bp deletion, pTandem-in19 expresses Cas9 plus in19-U1 and in19-D1 gRNAs creating a 179 bp deletion, and pTandem-in22 expresses Cas9 plus in22-U1 and in22-D1 gRNAs creating a 141 bp deletion. b) Deletions in CFTE cells using pTandem vectors analysed by agarose gel electrophoresis. "-" lanes indicate mock transfected cells (pcDNA3.1+ only), "+" lanes indicate cells transfected cells with pTandem-in12, -in19 or -in22. c) Sequence data from PCR amplicon generated from gel purified lower band in lane "22+" from (b). PAM sites are underlined and arrow indicates point at which manual deconvolution analysis of sequence begins. Three major targeted deletion products were identified: 1) DNA sequence consistent with both cut sites occuring 3 bp upstream of the PAM sequence; 2) DNA sequence consistent with proximal cut sites occuring 3 bp upstream of the PAM sequence and the with the distal cut sites occuring 4 bp upstream of the PAM sequence; and 3) DNA sequence consistent with both cut sites occuring 4 bp upstream of the PAM sequence.
(TIF)

S5 Fig Oligonucleotides used in this study. (PDF)
