# Supplementary figures and images for: Cas9/gRNA targeted excision of cystic fibrosis-causing deep-intronic splicing mutations restores normal splicing of CFTR mRNA
Source: PLoS One. 2017 Sep 1;12(9):e0184009. doi: 10.1371/journal.pone.0184009 (PMC5581164; doi:10.1371/journal.pone.0184009)

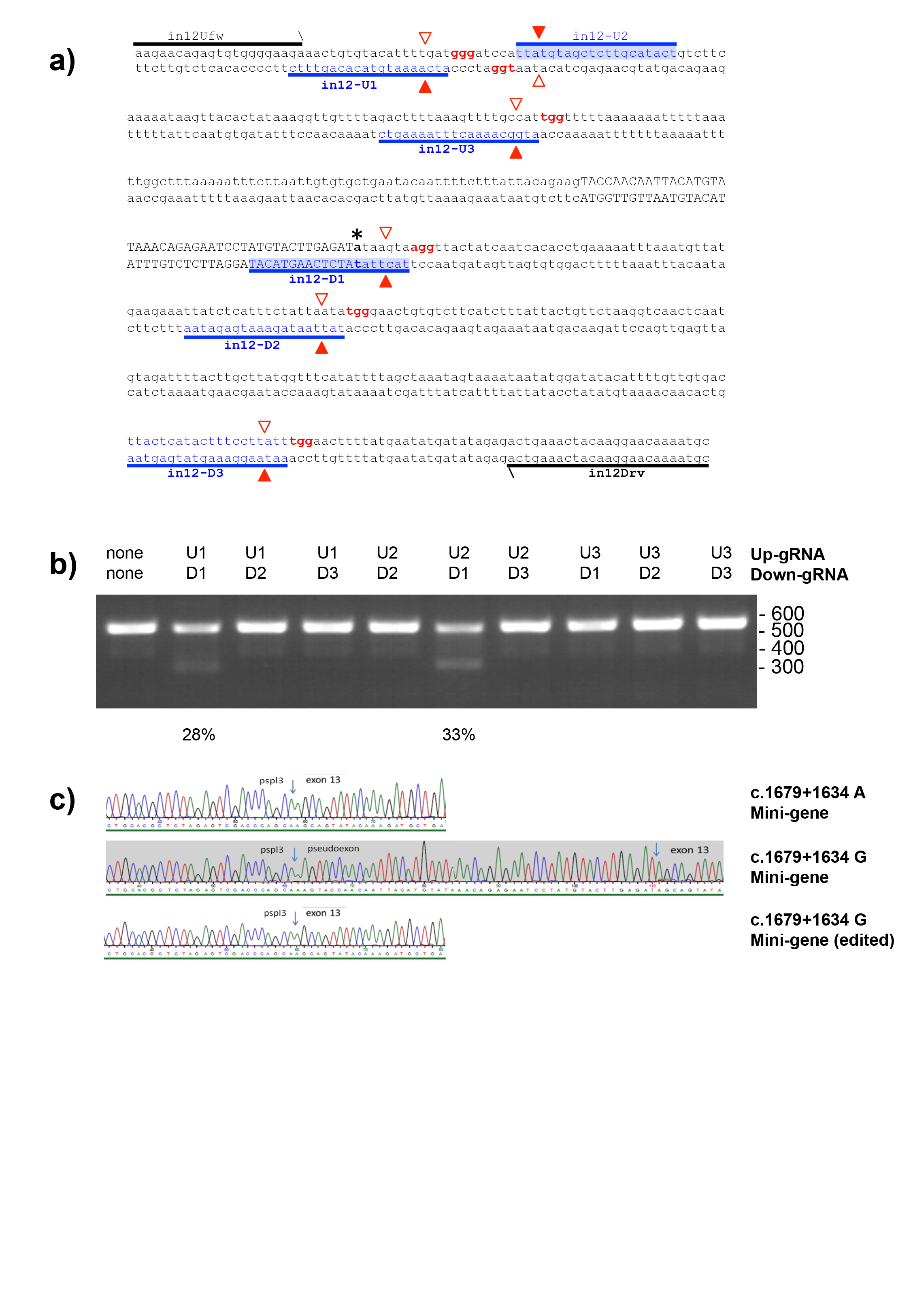

Supplement: S1 Fig — a) Sequence of 515 bp amplicon generated from intron 12 of CFTR using PCR primers in12Ufw and in12Drv with the 49 bp pseudoexon generated by the A>G mutation shown in UPPERCASE. gRNA target sites are underlined or overlined in blue according to orientation with PAM sequences (shown in red). The target sequences of the two gRNAs used in the pTandem-in12 vector are also shaded in blue. The predicted cut sites for all gRNAs are located 3 bp upstream of the PAM site (open triangles—RuvC site, filled triangles—HNH site). The predicted deletion created by pTandem-in12 is 203 bp. b) Agarose gel analysis of PCR amplicons generated from CFTE cells cotransfected with pairs of Cas9/gRNA expression plasmids. c) Sequence of main transcripts generated from pMGin12-WT (normal transcript), pMGin12-A>G (pseudoexon inclusion), and pMGin12-A>G co-transfected with pTandem-in12 (pseudoexon exclusion) in HEK293T cells. Arrows indicate junctions between exons. (TIF) [file pone.0184009.s001.tif]

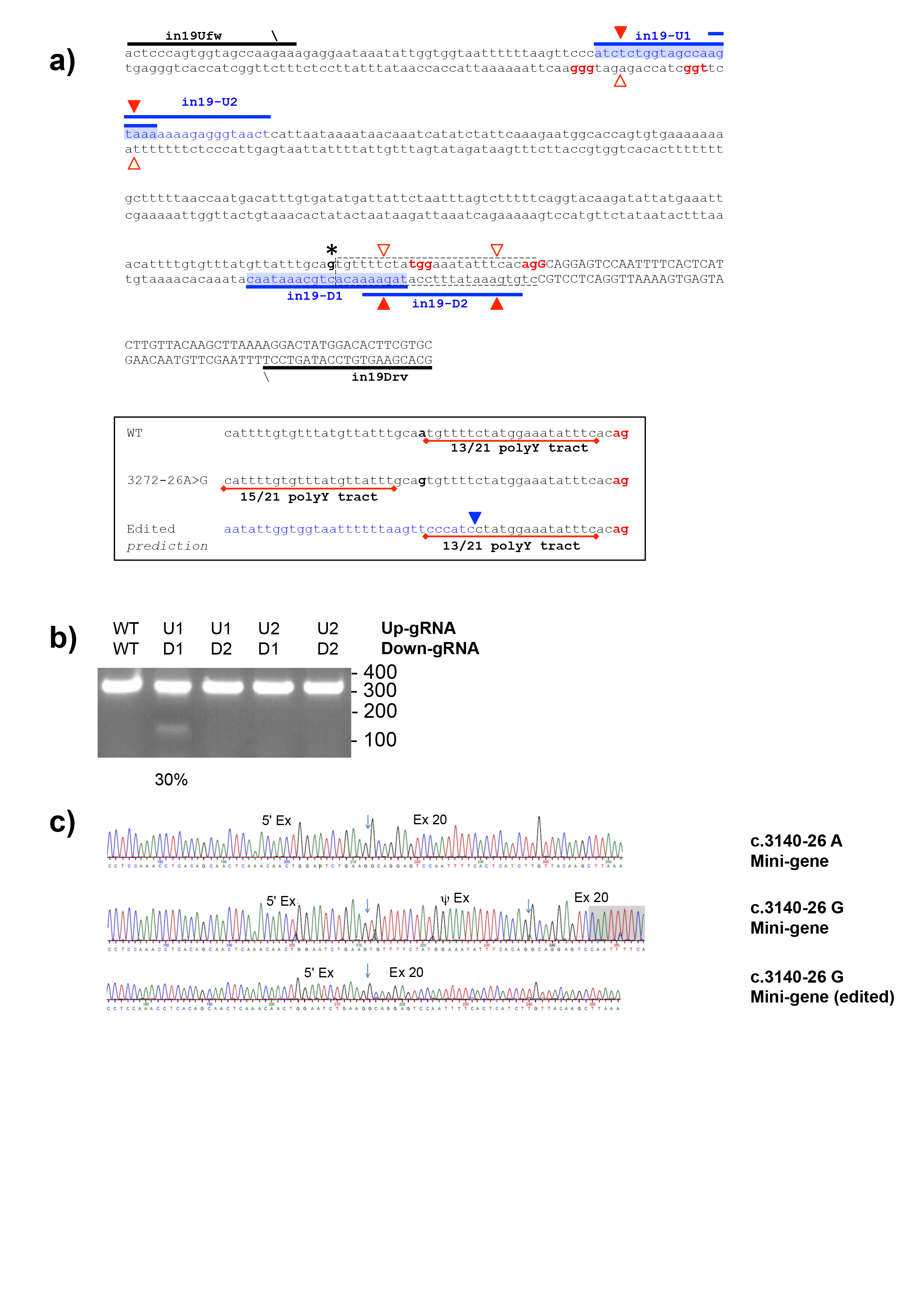

Supplement: S2 Fig — a) Sequence of 334 bp amplicon generated from intron 19 (lowercase)/EXON 20 (uppercase) using PCR primers inU19fw and in19Drv. gRNA target sites are underlined or overlined in blue according to orientation with PAM sequences (shown in red). The target sequences of the two gRNAs used in the pTandem-in19 vector are also shaded in blue. The predicted cut sites for all gRNAs located 3 bp upstream of the PAM site (open triangles—RuvC site, filled triangles—HNH site). The predicted deletion when using the pTandem-in19 vector is 179 bp. The inset shows predicted polypyrimidine tracts for wild-type intron 19 splice acceptor, c.3140-26A>G intron 19 splice acceptor and c.3140-26A>G intron 19 splice acceptor after targeted deletion. b) Agarose gel analysis of PCR amplicons generated from CFTE cells cotransfected with pairs of Cas9/gRNA expression plasmids. c) Sequence of main transcripts generated from pMGin19-WT (normal transcript), pMGin19-A>G (pseudoexon inclusion), and pMGin19-A>G co-transfected with pTandem-in19 (pseudoexon exclusion) in HEK293T. Arrows indicate junctions between exons. (TIF) [file pone.0184009.s002.tif]

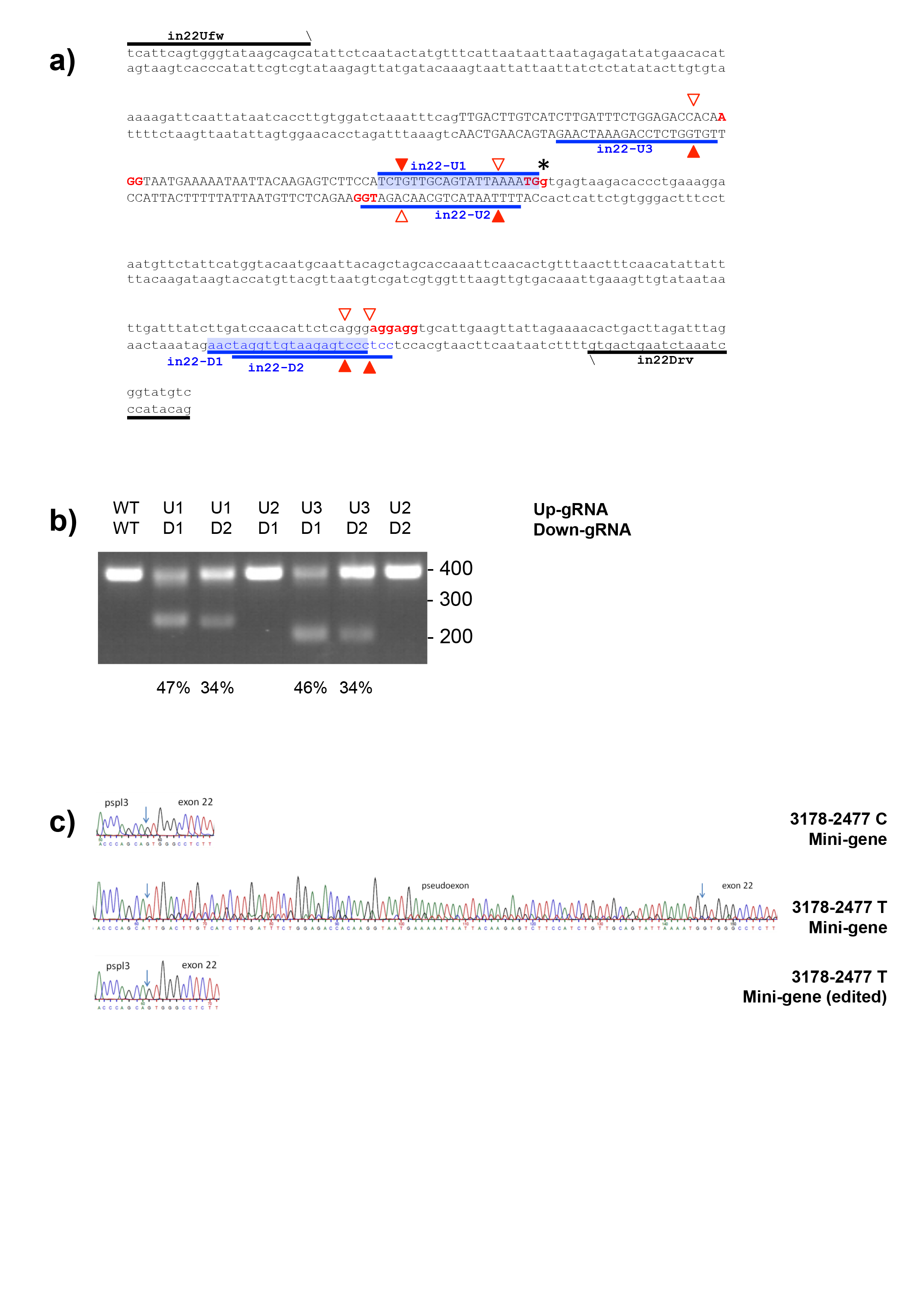

Supplement: S3 Fig — a) Sequence of 378 bp amplicon generated from intron 22 of CFTR using PCR primers in22Ufw and in22Drv with the 84 bp pseudoexon generated by the C>T mutation shown in UPPERCASE. gRNA target sites are underlined or overlined in blue according to orientation with PAM sequences (shown in red). The target sequences of the two gRNAs used in the pTandem-in22 vector are also shaded in blue. The predicted cut sites for all gRNAs are located 3 bp upstream of the PAM site (open triangles—RuvC site, filled triangles—HNH site). The predicted deletion created by pTandem-in22 is 141 bp. b) Agarose gel analysis of PCR amplicons generated from CFTE cells cotransfected with pairs of Cas9/gRNA expression plasmids. c) Sequence of main transcripts generated from pMGin22-WT (normal transcript), pMGin22-A>G (pseudoexon inclusion), and pMGin22-A>G co-transfected with pTandem-in22 (pseudoexon exclusion) in HEK293T. Arrows indicate junctions between exons. (TIF) [file pone.0184009.s003.tif]

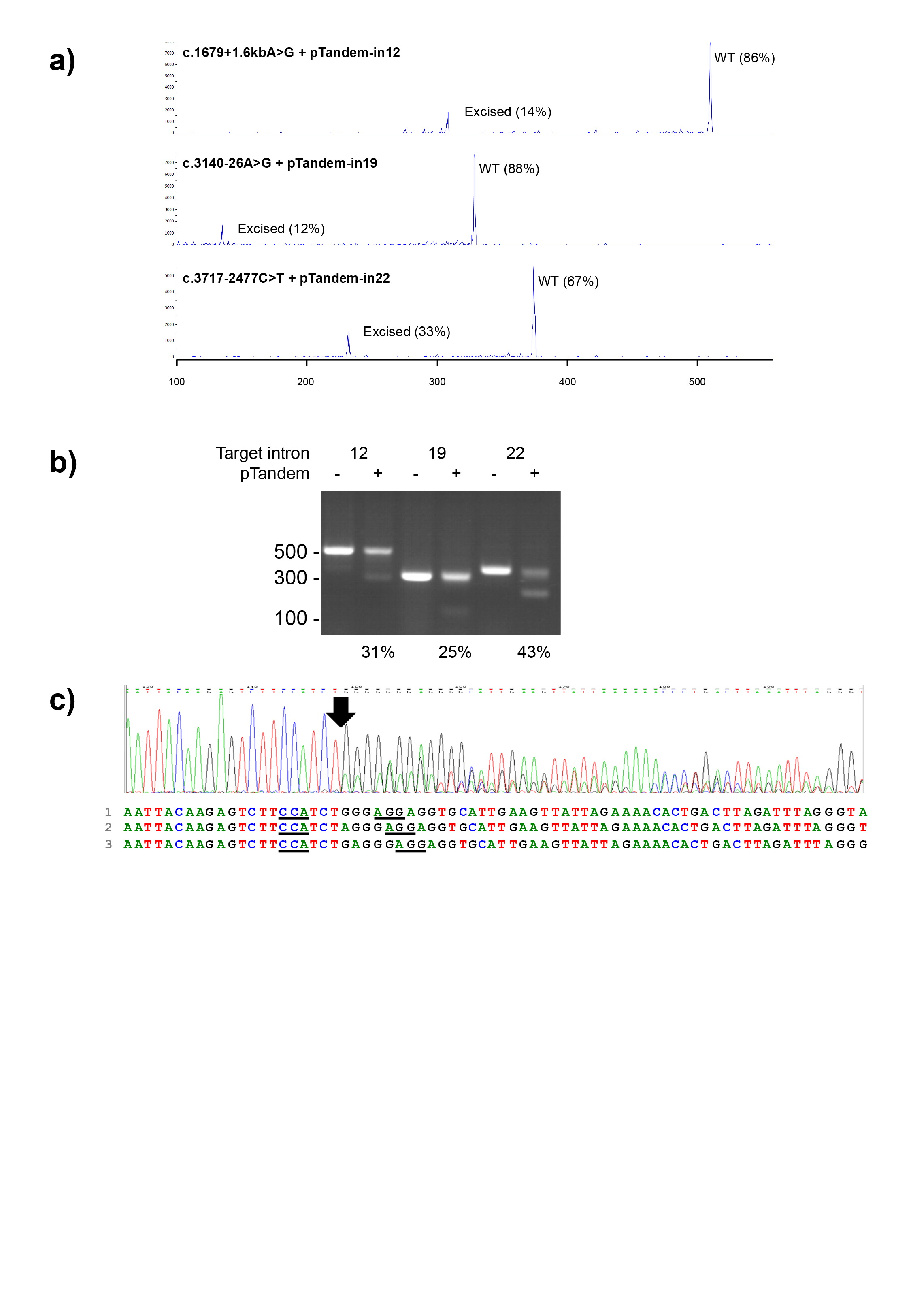

Supplement: S4 Fig — a) Deletions in CFTE cells using pTandem vectors anaysed by electropherogram. pTandem-in12 expresses Cas9 plus in12-U2 and in12-D1 gRNAs creating a 203 bp deletion, pTandem-in19 expresses Cas9 plus in19-U1 and in19-D1 gRNAs creating a 179 bp deletion, and pTandem-in22 expresses Cas9 plus in22-U1 and in22-D1 gRNAs creating a 141 bp deletion. b) Deletions in CFTE cells using pTandem vectors analysed by agarose gel electrophoresis. "-" lanes indicate mock transfected cells (pcDNA3.1+ only), "+" lanes indicate cells transfected cells with pTandem-in12, -in19 or -in22. c) Sequence data from PCR amplicon generated from gel purified lower band in lane "22+" from (b). PAM sites are underlined and arrow indicates point at which manual deconvolution analysis of sequence begins. Three major targeted deletion products were identified: 1) DNA sequence consistent with both cut sites occuring 3 bp upstream of the PAM sequence; 2) DNA sequence consistent with proximal cut sites occuring 3 bp upstream of the PAM sequence and the with the distal cut sites occuring 4 bp upstream of the PAM sequence; and 3) DNA sequence consistent with both cut sites occuring 4 bp upstream of the PAM sequence. (TIF) [file pone.0184009.s004.tif]
