# Supplementary material for: Cas9/gRNA targeted excision of cystic fibrosis-causing deep-intronic splicing mutations restores normal splicing of CFTR mRNA
Source: PLoS One. 2017 Sep 1;12(9):e0184009. doi: 10.1371/journal.pone.0184009 (PMC5581164; doi:10.1371/journal.pone.0184009)
Supplement: S5 Fig — (PDF) [file pone.0184009.s005.pdf]

**Oligo Name**	**Sequence (5’>3’)**	**Cloning site**
gRNA oligos cloning		
g-in12U-1fw	caccgGAAACTGTGTACATTTTGAT	
g-in12U-1rv	aaacATCAAAATGTACACAGTTTCc	
g-in12U-2fw	caccgAGTATGCAAGAGCTACATAA	
g-in12U-2rv	aaacTTATGTAGCTCTTGCATACTc	
g-in12U-3fw	caccgGACTTTTAAAGTTTTGCCAT	
g-in12U-3rv	aaacATGGCAAAACTTTAAAAGTCc	
g-in12D-1fw	caccgATGTACTTGAGATGTAAGTA	
g-in12D-1rv	aaacTACTTACATCTCAAGTACATc	
g-in12D-2fw	caccgTTACTCATACTTTCCTTATT	
g-in12D-2rv	aaacAATAAGGAAAGTATGAGTAAc	
g-in12D-3	caccgTTATCTCATTTCTATTAATA	
g-in12D-3rv	aaacTATTAATAGAAATGAGATAAc	
g-in19U-1	caccgTTTACTTGGCTACCAGAGAT	
g-in19U-1rv	aaacATCTCTGGTAGCCAAGTAAAc	
g-in19U-2	caccgAGTTACCCTCTTTTTTTACT	
g-in19U-2rv	aaacAGTAAAAAAAGAGGGTAACTc	
g-in19D-1	caccgGTTATTTGCAGTGTTTTCTA	
g-in19D-1rv	aaacTAGAAAACACTGCAAATAACc	
g-in19D-2	caccgTTTCTATGGAAATATTTCAC	
g-in19D-2rv	aaacGTGAAATATTTCCATAGAAAc	
g-in22U-1	caccgCATTTTAATACTGCAACAGA	
g-in22U-1rv	aaacTCTGTTGCAGTATTAAAATGc	
g-in22U-2	caccgCATCTGTTGCAGTATTAAAA	
g-in22U-2rv	aaacTTTTAATACTGCAACAGATGc	
g-in22U-3	caccgCTTGATTTCTGGAGACCACA	
g-in22U-3rv	aaacTGTGGTCTCCAGAAATCAAGc	
g-in22D-1	caccgTTGATCCAACATTCTCAGGG	
g-in22D-1rv	aaacCCCTGAGAATGTTGGATCAAc	
g-in22D-2	caccgATCCAACATTCTCAGGGAGG	
g-in22D-2rv	aaacCCTCCCTGAGAATGTTGGATc	
Minigenes cloning primers		
MGint12-int13FW	cacacactcgagTGTGTTGTCCAGTTTTGGATGA	*Xho*I
MGint12-int13RV	ctcacagctagcACTGGTTTAGCATGAGGCGG	*Nhe*I
MGint18-int20FW	ctcactctcgagTGACTAGGAATAGAATGGGGAGAG	*Xho*I
MGint18-int20RV	cacacagctagcACAATGGAAATTCAAAGAAATCACT	*Nhe*I
MGint22-int23FW	cacacactcgagACAGTACTGGATAGTCCTCTGA	*Xho*I
MGint22-int23RV	cacacatctagaGCCTATGAGAAAACTGCACTGG	*Xba*I

Directed Mutagenesis		

MD1811+1.6kbA>Gfw (c.1679+1634A>G)	CCTATGTACTTGAGATGTAAGTAAGGTTACTATC	
MD1811+1.6kbA>Grv (c.1679+1634A>G)	GATAGTAACCTTACTTACATCTCAAGTACATAGG	
MD3272-26A>Gfw (c.3140-26A>G)	GTGTTTATGTTATTTGCAGTGTTTTCTATGG	
MD3272-26A>Grv (c.3140-26A>G)	CCATAGAAAACACTGCAAATAACATAAACAC	
MD3849+10kbC>Tfw (c.3717-12191C>T)	CTGTTGCAGTATTAAAATGGtGAGTAAGACACCCTGAAAG	
MD3849+10kbC>Trv (c.3717-12191C>T)	CTTTCAGGGTGTCTTACTCaCCATTTTAATACTGCAACAG	
Sequencing Primers		
pspSEQfw	TCACAGTCTATTATGGGGTACGG	
pspSEQRV	AATTTCTGGGTCCCCTCCTGA	
pcas9SEQfw	GAGGGCCTATTTCCCATGATTCC	
pcas9SEQrv	GTCTGCAGAATTGGCGCAC	
pcas9tandemSEQfw	TTTGTGATGCTCGTCAGGGG	
pcas9tandemSEQrv	TGGAAAGTCCCTATTGGCGT	

Tandem gRNA Cloning		
pcas9TANDfw	CACACAGCTAGCGAGGGCCTATTTCCCATGATT	*Nhe*I
pcas9TANDrv	CACACATCTAGATCTAGCTCTAAAACAAAAAAGC	*Xba*I
Deletion Screening Primers		
in12Ufw	AAGAACAGAGTGTGGGGAAGA	
in12Drv	GCATTTTGTTCCTTGTAGTTTCAG	
in19Ufw	ACTCCCAGTGGTAGCCAAGA	
in19Drv	GCACGAAGTGTCCATAGTCCT	
in22Ufw	TCATTCAGTGGGTATAAGCAGCA	
in22Drv	GACATACCCTAAATCTAAGTCAGTG	
RT-PCR		
pspRTfw	TCTGAGTCACCTGGACAACC	
pspRTrv	ATCTCAGTGGTATTTGTGAGC	
RTex13rv	ACATCTAGGTATCCAAAAGGAGAGT	
RTex20rv	GCACGAAGTGTCCATAGTCCT	
